# *In Vivo* Ultrasonic Detection of Polyurea Crosslinked Silica Aerogel Implants

**DOI:** 10.1371/journal.pone.0066348

**Published:** 2013-06-14

**Authors:** Firouzeh Sabri, Merry E. Sebelik, Ryan Meacham, John D. Boughter, Mitchell J. Challis, Nicholas Leventis

**Affiliations:** 1 Department of Physics, University of Memphis, Memphis, Tennessee, United States of America; 2 Department of Otolaryngology, Head and Neck Surgery, University of Tennessee Health Science Center, Memphis, Tennessee, United States of America; 3 Department of Otolaryngology, Head and Neck Surgery, University of Tennessee Health Science Center, Memphis, Tennessee, United States of America; 4 Department of Anatomy and Neurobiology, University of Tennessee Health Science Center, Memphis, Tennessee, United States of America; 5 Department of Otolaryngology, Head and Neck Surgery, University of Tennessee Health Science Center, Memphis, Tennessee, United States of America; 6 Department of Chemistry, Missouri University of Science and Technology, Rolla, Missouri, United States of America; University of California, Berkeley, United States of America

## Abstract

**Background:**

Polyurea crosslinked silica aerogels are highly porous, lightweight, and mechanically strong materials with great potential for *in vivo* applications. Recent *in vivo* and *in vitro* studies have demonstrated the biocompatibility of this type of aerogel. The highly porous nature of aerogels allows for exceptional thermal, electric, and acoustic insulating capabilities that can be taken advantage of for non-invasive external imaging techniques. Sound-based detection of implants is a low cost, non-invasive, portable, and rapid technique that is routinely used and readily available in major clinics and hospitals.

**Methodology:**

In this study the first *in vivo* ultrasound response of polyurea crosslinked silica aerogel implants was investigated by means of a GE Medical Systems LogiQe diagnostic ultrasound machine with a linear array probe. Aerogel samples were inserted subcutaneously and sub-muscularly in a) fresh animal model and b) cadaveric human model for analysis. For comparison, samples of polydimethylsiloxane (PDMS) were also imaged under similar conditions as the aerogel samples.

**Conclusion/significance:**

Polyurea crosslinked silica aerogel (X-Si aerogel) implants were easily identified when inserted in either of the regions in both fresh animal model and cadaveric model. The implant dimensions inferred from the images matched the actual size of the implants and no apparent damage was sustained by the X-Si aerogel implants as a result of the ultrasonic imaging process. The aerogel implants demonstrated hyperechoic behavior and significant posterior shadowing. Results obtained were compared with images acquired from the PDMS implants inserted at the same location.

## Introduction

Design, development, and characterization of porous biomaterials is a rapidly growing area of materials science and has a broad range of applications in biomedicine and, the medical device industry [1], [2], [3], [4], [5], [6], [7], [8], [9]. Solid porous biomaterials have been developed using a variety of methods and techniques such as electrospinning, particle sintering, foaming, and sol-gel [10], [11], [12], [13], [14]. Each method of material fabrication gives rise to distinct physical and chemical features with a broad range of properties to choose from. Since different cell types require different architectures for the promotion or inhibition of adhesion, proliferation, and differentiation, each technique serves a different application window.

An important part of the design and development of new biomaterials is the ability to image and locate the inserted material after implantation with minimum harm to the patient. Implant tracking and imaging is particularly important in applications where the implant is designed to degrade over time, or, concerns regarding implant travel exist. Integration of implants with nearby tissue is also an important aspect that can be studied if a non0invasive imaging technique exists. Imaging techniques used today include MRI, X-ray, IR, CT scan, and, ultrasound-based detection methods [15], [16], [17], [18], [19], [20], [21], [22], [23], [24]. Among these techniques sound-based detection is the most versatile, innocuous, and inexpensive method currently available for diagnostic and therapeutic studies since it does not require any patient preparation and can be performed with relative ease. There is increasing evidence that patients prefer ultrasound imaging to other techniques such as MRI, X-ray, and CT scan since there is no need for invasive procedures, injection of contrasting medium, or exposure to harmful ionizing radiation [18].Furthermore, the ability to visualize tissue in real-time motion and the superior resolution of highly organized tissue such as a tendon [18] makes ultrasound imaging a valuable tool.

The ultrasonic properties of materials and the nature of their interaction with the incoming ultrasound wave affect the quality, clarity, sharpness, and accuracy of the ultrasound image formed during sonography. This interaction is reflected in the acoustic impedance of the material of interest which is derived from the mass density of the material and the velocity of the acoustic wave in that material [25], [26]. The wave velocity is in turn affected by the material stiffness and its elastic modulus [26]. Soft biological tissue is typically modeled as a fluid [16] giving rise to acoustic impedance values in the range of 160–165 KRayl [25] while stiff porous solids such as bone have impedance values of the order of 780 KRayl [25]. Table 1 lists typical values for bulk density and wave velocity for common biological and biomedical materials and the corresponding acoustic impedance values.

**Table 1:** Comparison of typical acoustic impedance values for common biological and biomedical materials.

Material	Impedance Z (KRayl)	Velocity V (m/s)	Density ρ (g/cm^3^)
Air^25^	0.04	330	0.0013 (at STP)
Blood^25^	161	1570	1.04
Soft tissues (avg)*	163	1540	1.01–1.06
Muscle^25^	170	1580	1.05
Bone†	780	4080	1.5–2.0
Fat^25^	138	1450	0.94
Water^25^	148	1480	1.0
UHMWPE^25^	194	2000	0.94
Stainless steel^25^	4576	5800	7.93
Silicone (PDMS)∧	150	1300	1.5
Aerogel (native)^29^	N/A	120–310	0.071–0.285

† Physics of the Human Body Irving P. Herman Chapter 7 Springer 2008.

* Yamauchi T, Yanai M, Takahashi S, Man NK (1996) Blood density monitoring during dialysis. Artif Organs. 9∶981–985.

∧ Oppenheim IJ, Jain A, Greve DW (2003) MEMS Ultrasoinc Transducers for the Testing of Solids. IEEE transactions on Ultrasonics, Ferroelectrics, and Frequency Control 50∶305–311.

Polyurea crosslinked silica aerogels (X-Si aerogel) [27] are highly porous open-pore solids developed by means of the sol-gel technique with unique and adjustable properties attractive to the biomedical industry. With pore sizes typically less than 300 nm, aerogels offer superior electric, thermal, and acoustic insulating capabilities [28], [29], [30], [31], [32] attractive to a variety of industries and applications. Recent *in vivo* [33] and *in vitro* [34], [35] studies have demonstrated short and long term biocompatibility of this type of aerogel, paving the way for further understanding of the behavior of this material in a biological and physiological environment. The highly porous and mechanically strong nature of X-Si aerogel creates well-defined acoustic and ultrasonic characteristics that can be utilized for live imaging of aerogel-based implants and medical devices.

In this study, we present the first evidence that *in vivo* polyurea crosslinked silica aerogel implants can be imaged ultrasonically. A General Electric Medical Systems LogiQe diagnostic ultrasound machine was used to image X-Si aerogel implants inserted sub-muscularly and subcutaneously in a) freshly euthanized Sprague-Dawley rat and b) human cadaver at a 13 MHz setting-significantly below the cavitation frequency [36]. Aerogel implants showed strong contrast compared to neighboring soft tissue and appeared isoechoic while polydimethylsiloxane (PDMS) control material demonstrated hypoechoic behavior under similar conditions. Crosslinked aerogel implants were easily identified and the implant dimensions inferred from the images matched the physical size of the implants. The aerogel implants also demonstrated some hyperechoic behavior and significant posterior shadowing.

## Materials and Methods

### 2.1 Synthesis of Aerogels

**Figure 1:**
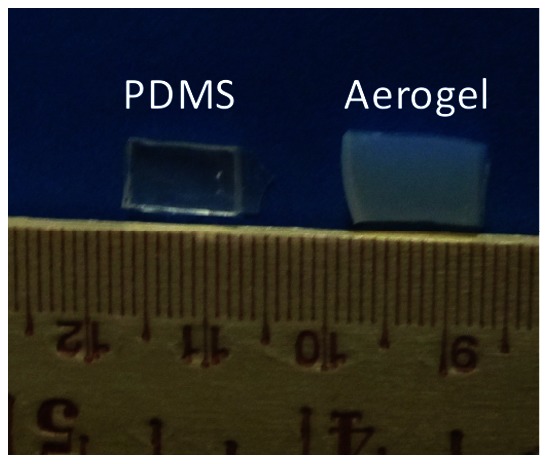
PDMS and X-Si aerogel implants prior to implantation in Sprague-Dawley rat. Optical image of Sylgard 184 PDMS and X-Si aerogel samples prior to insertion in Sprague-Dawley rat for ultrasonic imaging.

Two solutions, the first containing 3.85 mL tetramethoxysilane (TMOS) and the second one containing 4.5 mL methanol and 1.5 mL water as well as a 25 mL of 3-aminotrioxypropysilane were mixed in a 250 mL beaker with a sterile glass stirring rod. The resulting sol (colloidal suspension) was immediately poured into cylindrical molds and gelled within 60 sec while still cold. The gels were aged for 3 hrs in a methanol bath and subsequently washed with methanol (once) and four times with acetonitrile, using 4–5 times the volume of the gel for each wash. Subsequently, gels were transferred to an isocyanate bath containing 33 g of Desmodur N3200 (Bayer) in 94 mL of acetonitrile. The volume of the bath was again 4–5 times the volume of each gel. After 24 hrs, the gels were transferred to fresh acetonitrile and they were heated at 70°C for 72 hrs in a Blue-M Therm oven. At the end of the period, the gels were washed another four times with fresh acetonitrile (24 hrs each time) and then were dried by means of critical point drying, using liquid CO_2_ in a Polaron E3000. A Table top Buehler slow speed saw was used for cutting samples into smaller geometries prior to implant procedure (Figure 1). Pigmented X-Si aerogel samples were synthesized according to previously established recipe [37].

### 2.2 Synthesis of Sylgard 184 PDMS

Clear Sylgard 184 samples were prepared according to the guidelines provided by the manufacturer(Dow Corning, Midland, MI) i.e. a ratio of 10∶1 elastomer prepolymer (A) to crosslinker (B). The A and B components were thoroughly mixed in a Pyrex container with a metallic spatula. The mixture was completely outgassed in a Precision Scientific No. 6500 vacuum oven for approximately 2 hrs until the mixture had no air bubbles remaining. The outgassed mixture was then poured slowly into square aluminum molds. The molds were then transferred to the vacuum oven, out-gassed one more time, and finally heat cured at 80°C for 24 hrs while under vacuum. Polymer samples were cut to dimensions similar to the X-Si aerogel sample prior to the implanting stage (see Figure 1).

### 2.3 Imaging Procedure-female Sprague-Dawley Rat

**Figure 2:**
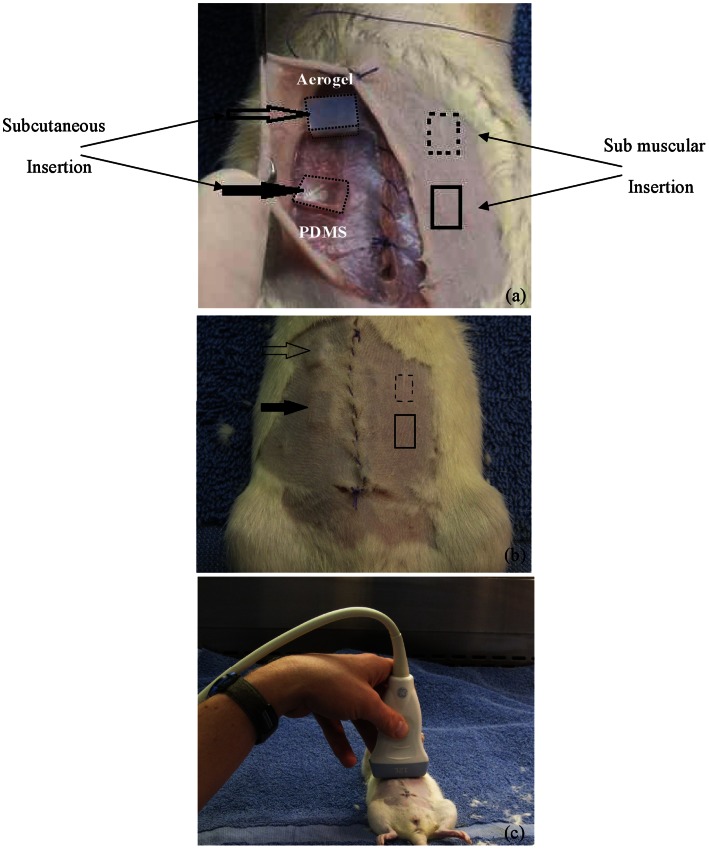
Subcutaneous and sub muscular implant insertion in Sprague-Dawley rat. Optical images of (a) X-Si aerogel and Sylgard 184 implants being positioned in the abdominal region of a female Sprague-Dawley rat subcutaneously and sub-muscularly. (b) Abdominal section of rat sealed, arrows indicating left and right abdominal positions of four implants. (c) Ultrasound probe positioning during scanning and acquisition of images.

A 150 g female Sprague-Dawley rat was selected for *in vivo* analysis of the ultrasound properties of the PCSA implant and the polydimethylsiloxane (PDMS) polymer-Sylgard 184 (Dow Corning, Midland, MI) used for comparison. The rat was sacrificed with an overdose of CO_2_ immediately before implant surgery. With the rat in the supine position, the abdomen was shaved and a midline vertical incision was made from the xiphoid process inferiorly down to the pelvic inlet. A subcutaneous plane was created on the right half of the abdomen and a submuscular plane was created on the left half of the abdomen. A X-Si aerogel implant was inserted superiorly and a Sylgard 184 implant was inserted inferiorly on each side of the abdomen within the newly created pockets as shown in Figure 2a. The muscular layers and cutaneous layers were then reapproximated with an absorbable suture (Figure 2b). Ultrasound images of all four implants were obtained in a longitudinal (vertically oriented) view with a 13 MHz linear array probe generated by a General Electric Medical Systems LogiQe (Healthcare, Wauwatosa, Wisconsin) diagnostic ultrasound machine fitted with a 7.5- to 13-MHz linear array transducer (Figure 2c).This study was approved by the Animal Care and Use Committee at the University of Memphis.

### 2.4 Imaging Procedure-Human Cadaver

**Figure 3:**
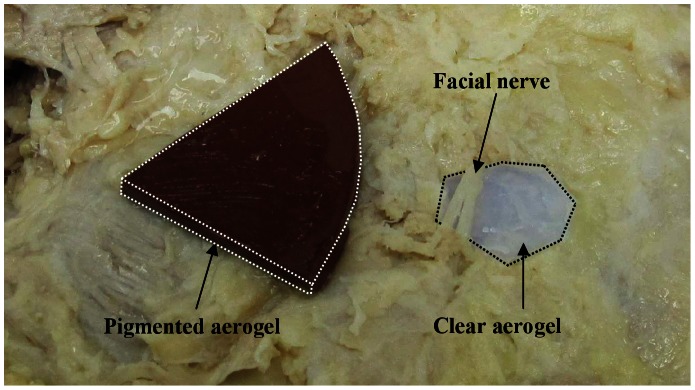
Pigmented and clear X-Si aerogel implants inserted subcutaneously in a human cadaver. Pigmented and clear X-Si aerogel implants positioned subcutaneously on the right cheek of human cadaver near and immediately under the bucal nerve region, before repositioning the skin flap.

A human cadaver fixed in a solution of 75.68% isopropanol, 18.92% dipropylene glycol, and 5.4% formalin was used for this section of the study. The skin over the parotid region (including some subcutaneous tissue) was reflected laterally, and a buccal branch of the facial nerve was dissected free of the surrounding parotid gland tissue. Precut segments of pigmented and clear X-Si aerogel implants were placed on the right cheek of the cadaver (Figure 3) and in one case under the buccal nerve branch. The skin flap was placed back over the implants (and the entire region) and the ultrasound probe described previously was applied to the exterior surface of the skin. Ultrasound images of both implants were recorded at a frequency setting of 13 MHz longitudinally.

Data collection from the human cadaver qualified for exempt status by the University of Tennessee Health Science Center Institutional Review Board under Federal Regulations 45 CFR 46.102(f) definition of “Human Subjects”. The cadaver was collected from the Department of Anatomy & Neurobiology at UTHSC, via the anatomical bequest program. All the cadavers were donors. All donors sign a form that confirms that the donations may be used for scientific research on the donation form.

## Results and Discussion

### 3.1 Imaging in a Hydrated Environment-Rat Model

#### Subcutaneous

**Figure 4:**
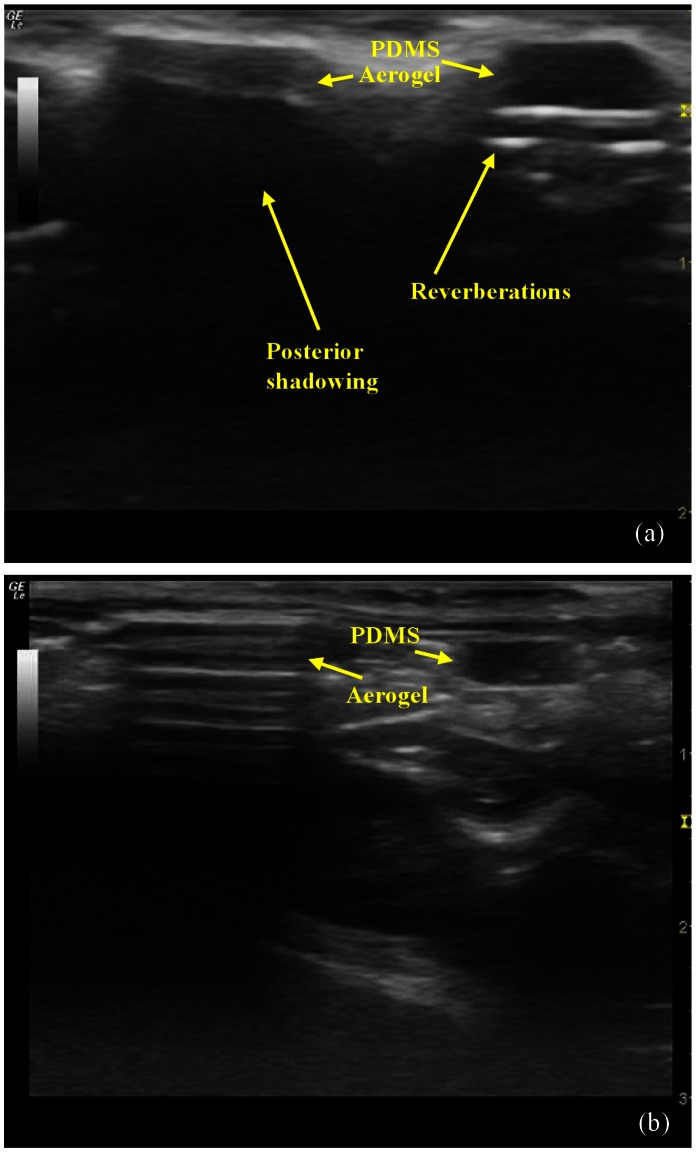
Ultrasonic images of subcutaneous and sub muscular implants in Sprague-Dawley rat at 13 MHz. Ultrasound images of Sylgard 184 and X-Si aerogel acquired from (a) subcutaneous and (b) submuscular abdominal implantation sites. Strong attenuation by X-Si implants has lead to significant posterior shadowing (indicated by arrow) while minimum attenuation by PDMS has created image aberrations (indicated by arrow) referred to as reverberations. Images reflect accurately the size and shape of the implants at both locations.

Clear X-Si aerogel and Sylgard 184PDMS implants inserted subcutaneously were easily identifiable compared to the surrounding soft tissue when scanned at the13 MHz setting with a linear array probe (Figure 4a). Aerogel implants inserted subcutaneously appeared isoechoic, homogeneous, with some posterior shadowing. The region immediately below the implant appeared dark confirming strong ultrasound attenuation expected of aerogels and their role as a highly attenuating medium. Some echogenicity was also observed surrounding the aerogel implant. The hyperechoic behavior particularly at the boundary between the aerogel implant and the soft tissue is attributed to the strong impedance mismatch between the soft tissue and the aerogel implant. In order to calculate the acoustic velocity v and acoustic impedance Z of X-Si aerogel used in this study it was assumed that the propagation of ultrasound waves in aerogels is via the skeleton and not the medium within the porous system [29]. The ultrasound wave propagation was also treated as a one-dimensional (1D) problem where incident, reflected, and transmitted waves were all assumed to be normal to the interface. The acoustic velocity v and acoustic impedance Z of X-Si aerogel were then calculated using Equation 1 [31], [25]:
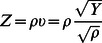
(1)Where Y is the stiffness (modulus) and ρ is the mass density of the material. For a stiffness value of 120 MPa [27] and a density of 0.4 g/cm^3^, an acoustic velocity of 547 m/s was calculated which lead to an impedance value of 2.2×10^5 ^kgm^−2^ s^−1^ or 22 KRayl for the X-Si aerogel implant.

Since aerogel-based implants are expected to be used primarily in the peripheral regions of the body (at depths shallow in comparison to the bony structure of the anatomy) it is unlikely for aerogel-based implants to interfere with imaging of skeletal structure. Also, given that the propagation of sound in a porous material is governed mainly by physical characteristics such as porosity (*φ*) and tortuosity (*q*) [38], aerogels and bone could have distinctly different appearances. The impedance value of 22 KRayl calculated here for a X-Si aerogel implant is significantly different when compared to acoustic impedance values of common *in vivo* materials such as those listed in Table 1, further suggesting that such implants will be distinguishable when compared to other materials, in particular bone.

The subcutaneous Sylgard 184 implant on the other hand appeared hypoechoic and homogeneous, characteristics of a low attenuating medium (see Figure 4a),similar to features seen in ultrasound images of cartilage and silicone-based implants. In the case of the Sylgard 184 PDMS implant aberrations in the form of bright bands (indicated by the arrow, Figure 4a) were detected immediately below the implant and echoes received from points distal to this material were higher in intensity than echoes received from a similar depth in the imaging plane, confirming that they are indeed artifacts.

#### Sub-muscular

In the submuscular location, the X-Si aerogel implant appears more hypoechoic with posterior shadowing, indicating its deeper location (Figure 4b). The Sylgard 184 implant, despite being submuscular, retained imaging characteristics similar to the subcutaneous placement due to the depth. Despite the change in echogenicity with depth, the geometric identity is retained. In all cases, the ultrasound images reflect accurately the shape, size, and depth of insertion of both types of implants.

#### 3.2 Imaging in a cadaveric environment-Human cadaver model

**Figure 5:**
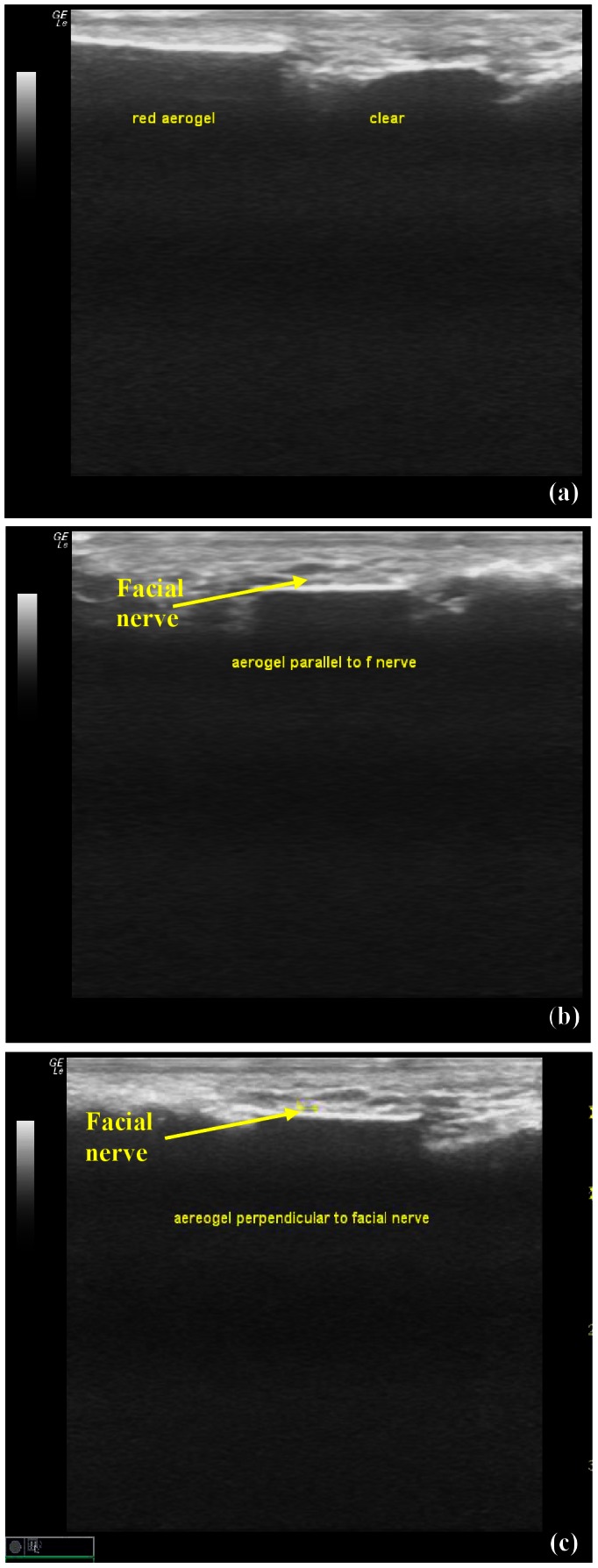
Ultrasonic imaging of subcutaneous X-Si aerogel implants in human cadaver at 13 MHz. (a) Ultrasonic images of pigmented and clear X-Si aerogel implants positioned immediately under the skin, in the parotid region. Some posterior shadowing is observed. The structure of the pigmented (red) and clear aerogels were indistinguishable at the imaged frequency. (b) Parallel and (c) perpendicular orientations of clearX-Si aerogelwith respect to the facial nerve, as indicated by the arrows.

Both pigmented and clear aerogel implants appeared isoechoic and homogeneous (see Figure 5a) but with less posterior shadowing than in the immediate post-euthanized rat discussed in section 3.1. At the imaged frequency the pigmented and clear aerogels did not display noticeable differences except for obvious geometrical differences. Once again, the ultrasound images reflect accurately the shape, size, and depth of insertion of the implants. The clear X-Si aerogel was imaged both in directions parallel (Figure 5b) and perpendicular (Figure 5c) to the facial nerve, positioned immediately above the implant, as indicated by the arrows. Table 2 summarizes the ultrasonic responses of X-Si aerogel implants as well as Sylgard 184 implants under various conditions, as discussed in this work.

**Table 2:** Summary of the ultrasonic response of implants imaged under various conditions.

	Material	Implant site	Response	Frequencysetting (MHz)
Rat Model- Abdominalregion	Sylgard 184	Subcutaneous	Hypoechoic/Reverberations present	13
	Sylgard 184	Submuscular	Hypoechoic	13
	Clear X-Si aerogel	Subcutaneous	Hyperechoic/Isoechoic/Strong Posterior shadowing	13
	Clear X-Si aerogel	Submuscular	Moderately Hypoechoic	13
Cadaver Model- Facialregion	Pigmented X-Si aerogel	Subcutaneous	Echogenic/Homogeneous	13
	Clear X-Si aerogel	Subcutaneous	Echogenic/Homogeneous	13

### Summary and Conclusion

The ultrasonic behavior of X-Si aerogels were investigated *in vivo* both in human cadaveric and freshly euthanized animal models. The unique properties of X-Si aerogels have been successfully utilized for *in vivo* imaging at various insertion depths. This study demonstrates the strong potential of aerogel-based materials and scaffolds as a future biomedical material.
